# Nitrogen acquisition and resource allocation strategies in temperate seagrass *Zostera nigricaulis*: Uptake, assimilation and translocation processes

**DOI:** 10.1038/s41598-018-35549-3

**Published:** 2018-11-21

**Authors:** S. Nayar, M. G. K. Loo, J. E. Tanner, A. R. Longmore, G. P. Jenkins

**Affiliations:** 1 South Australian Research and Development Institute – Aquatic Sciences, West Beach, South Australia Australia; 2 Centre for Aquatic Pollution Identification and Management, University of Melbourne, Victoria, Australia; 3 School of BioSciences, University of Melbourne, Victoria, Australia; 4 School of Life and Environmental Science, Deakin University, Victoria, Australia

## Abstract

The dominant seagrass in Port Phillip Bay (PPB), Australia, *Zostera nigricaulis*, declined between 2000 and 2011, coinciding with the ‘Millennium drought’ that ended in 2009. These seagrasses are nitrogen-limited, underpinning the need to develop nitrogen budgets for better ecosystem management. Environmentally realistic measurements of specific uptake rates and resource allocation were undertaken to develop nitrogen budgets and test the hypothesis that the above-ground and below-ground compartments are able to re-mobilise ammonium and nitrate through uptake, translocation and assimilation to adapt to varying levels of nitrogen in the ecosystem. Uptake of ^15^N labelled ammonium and nitrate by above- and below-ground seagrass biomass, epiphytes and phytoplankton was quantified in chambers *in situ*. Preferential uptake of ammonium over nitrate was observed, where the uptake rate for nitrate was about one sixth of that for ammonium. Epiphytes and phytoplankton also registered an increased affinity for ammonium over nitrate. Translocation experiments demonstrated the uptake by both the above-ground and below-ground biomass, respectively from the water column and pore water, and subsequent translocation to the opposite compartment. Acropetal translocation (below- to above-ground biomass) was more prevalent than basipetal translocation. This is a unique outcome given basipetal translocation has been widely reported for *Zostera* by other researchers.

## Introduction

Seagrasses are highly productive marine angiosperms that thrive in shallow coastal waters^1^ and provide critical habitats and a nutritional base for finfish, shellfish, and herbivorous animals^2^. Coastal urbanisation and nearshore developments have resulted in declines in water quality affecting seagrasses^3,4^. Such activities, in recent decades, have resulted in increased nutrient loading and turbidity in nearshore systems dominated by seagrasses^3,5,6^, affecting the distribution and composition of seagrass meadows^7–10^. Atypical of the seagrasses in the current study, excessive nitrogen loading globally has been reported to have detrimental effects on seagrass-dominated estuaries by inhibiting seagrass growth and survival through the stimulation of phytoplankton, epiphytic algae and benthic microalgal growth^11–13^. Eutrophication is also considered to be a major cause of the loss of seagrass in Australia^14–16^. Eutrophication not only has an indirect effect by stimulating algal overgrowth and consequently reducing available light, but for some species a direct physiological effect^8,13,17^.

Various studies have identified ammonium (NH_4_^+^), nitrate (NO_3_^−^) and nitrite (NO_2_^−^) as the largest sources of nitrogen for seagrass^13,18^. There is limited knowledge on uptake rates of organic nitrogen sources in seagrass beds, but evidence in the literature indicates they are relatively insignificant if inorganic nitrogen is prevalent^19,20^. Since nitrate and ammonium are regarded as the most significant sources of nitrogen, most studies assume these to be the only source^13^. Published evidence suggests this to be a valid assumption, with nitrate and ammonium supplying over 90% of external nitrogen to seagrass^13,21^.

Sediment nitrogen pools supply the majority of nitrogen to rooted marine plants; therefore, nutrient cycling in sediments is a critical process. Unlike various species of algae that are dependent exclusively on nutrient concentrations in the water column, seagrasses are rooted plants, that can obtain majority of their nutrient requirement from the sediment or substrate^22^. Seagrasses are therefore capable of recycling nutrients in the ecosystem that would otherwise be trapped in the sediment and become unavailable. Although sediment pore water is generally regarded to be the primary nitrogen source for seagrass, the evidence suggests that uptake of both nitrogen and phosphorus by below-ground biomass is insufficient to meet the total nutrient requirement of the seagrass^21,23^. Some species, such as *Amphibolis antarctica* and *Phyllospadix torreyi*, that are commonly found on rocky substrates and have little or no sediment around the roots, obtain the majority of their nutrient demands from the water column by uptake through leaves^24,25^. Young, actively growing roots have been reported to account for most of the nitrogen taken up in *Thalassia hemprichii* and *Zostera marina*^23,26^. Another important mechanism in seagrass nitrogen dynamics is the process of translocating nitrogen from the rhizomes and roots to the leaves and vice versa, depending on the uptake mechanism prevalent. Although the existence of internal translocation of nitrogen between the above- and below-ground compartments has been acknowledged, there is a paucity of information quantifying the distribution of nitrogen between these compartments together with uptake rates^27^.

While nutrient dynamics, uptake and resource allocation are well documented in tropical seagrasses, there is less understanding in temperate oligotrophic systems. The present study is therefore significant as there is a paucity of information on the assimilative capabilities of seagrasses found in these regions, where a comparatively small increase in nutrient load, particularly nitrogen, exerts a far greater influence on the health of seagrass than those found in mesotrophic systems. Such studies are fundamental to further our understanding of the influence of nutrients on biological productivity in pristine and impacted systems.

Historical trends in the Port Phillip Bay (PPB) suggest that both nutrient availability and sediment movement determine the distribution and cover of seagrasses. Based on aerial photography, seagrass cover along the southern margins of PPB increased between 1960 and the 1990s, declining rapidly from the late 1990s onwards. This decline in seagrass cover coincided with a prolonged period of drought in southern Australia (1998–2009). Over the past decade >90% of seagrass cover has disappeared in certain regions of PPB. Higher seagrass cover in PPB appears to be correlated historically with higher nutrient loadings from the WTP and surrounding catchments, suggesting that nutrient availability may limit the growth of seagrasses in some regions of PPB. This is in stark contrast to reduced seagrass health in other parts of PPB resulting from localised nutrient run-off leading to elevated epiphyte growth, burial of seagrass due to sand movement, low reproduction success and other physical processes such as wave energy and light availability^28–30^.

The present study therefore quantified the uptake and translocation rates of ammonium and nitrate in *Zostera*, a dominant seagrass in PPB^31^. This study quantified specific uptake rates by above-ground seagrass biomass, below-ground seagrass biomass, epiphytic algae and phytoplankton using stable isotope (^15^N) labelled ammonium and nitrate *in situ*. This study was undertaken to obtain environmentally realistic uptake data to develop nitrogen budgets and to test the hypothesis that the above-ground and below-ground compartments of *Zostera nigricaulis* are able to remobilise ammonium and nitrate through uptake, translocation and assimilation from the above-ground compartment to the below-ground compartment and vice versa as a mechanism to adapt to nitrogen availability.

The study is unique in that the quantification of specific uptake rates of inorganic nitrogen by the above-ground seagrass biomass, below-ground seagrass biomass, epiphytic algae and phytoplankton were undertaken simultaneously with studies to quantify translocation rates between the above- and below-ground compartments. As the specific uptake rates and translocation studies for ammonium and nitrate were undertaken at the same time at each of the three study sites, it allows comparison of the two nitrogen sources. Given these experiments were conducted *in situ* under environmentally realistic conditions, the use of the data in whole ecosystem models will enable managers to better understand ecosystem processes to assist with better management of the seagrass ecosystems in PPB.

## Methods

### Field location and ambient environment

#### Sampling location

The present study was undertaken in PPB (38°S, 148°E), a large, shallow, semi-enclosed marine embayment with a water spread and catchment area of 1,950 km^2^ and 9,790 km^2^, respectively. The Bay forms a major resource for Melbourne, Australia’s second largest city, with a population of ~3.7 million people. *Zostera* is the dominant seagrass in the PPB accounting for about 95% of the total seagrass (60 km^2^ in areal cover), followed by *Amphibolis* and *Halophila* at 3% and 2%, respectively^32,33^. About 95% of the seagrasses in the Bay occur in waters shallower than 5 m, where there is good access to light^34^.

**Figure 1:**
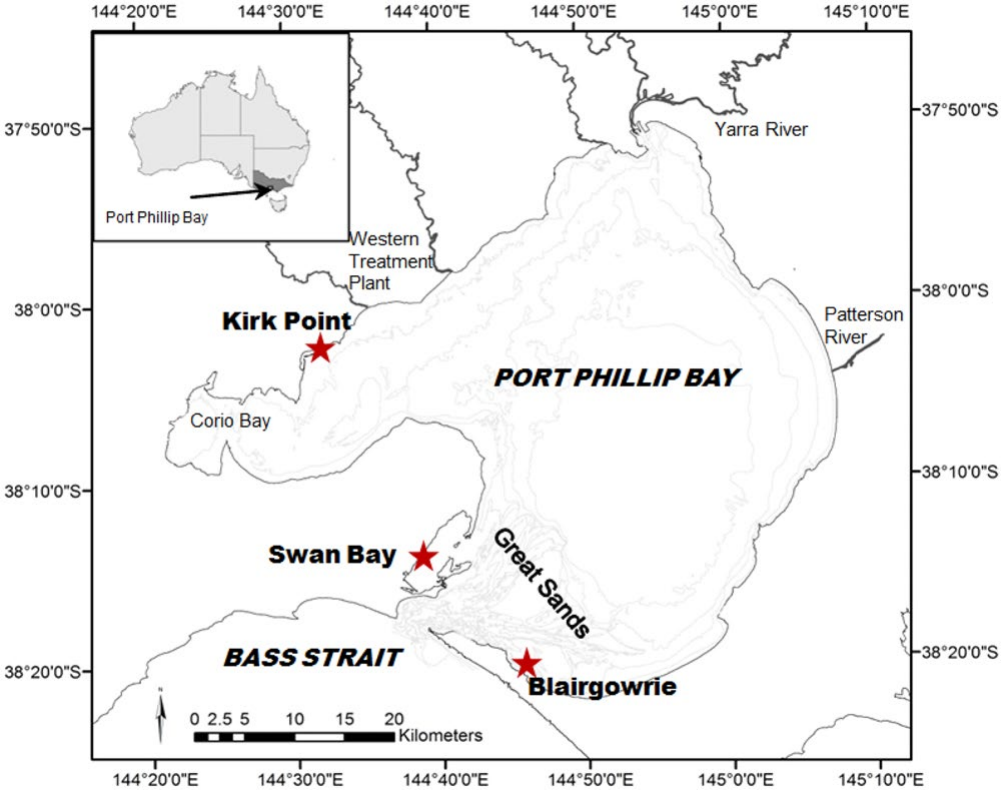
Location of the sampling sites where the chamber deployments were carried out in Port Phillip Bay. Base map was created using ArcMap.

Preliminary trials undertaken by DPI Victoria on ^15^N background signatures of *Zostera nigricaulis* beds led to the selection of three sites for chamber deployments in PPB, Victoria. The chosen sites were Blairgowrie (BG), Swan Bay (SB) and Kirk Point (KP; Fig. 1). The three study sites were chosen based on the distinct δ^15^N profiles in seagrass as established from baseline studies^29,35^, viz., 7, 0 and 17‰ at BG, SB and KP, respectively. The three study sites comprised beds of *Zostera nigricaulis* with an average water depth of about 2 m during high tide. Both nutrient uptake and translocation chamber deployments were carried out between 11^th^ and 13^th^ December 2012, commencing at 1030 h each day. Nutrient uptake experiments for plankton at Kirk Point could not be completed due to the disruption of the experiment by a squall resulting in the loss of samples.

#### Collection of background samples

All background sample collections were undertaken at each of the three study sites well before the chamber deployment. Approximately 2 L of water was collected using a Van Dorn sampler about 0.5 m above the seagrass bed for determination of background levels of: (a) ^15^N in phytoplankton and bacteria (suspended particulates); (b) qualitative and quantitative analysis of phytoplankton; and (c) measurement of ambient water quality.

Immediately upon sampling, about 50 mL of the water was filtered using a syringe filter (0.2 μm pore size) and transferred into labelled, pre-rinsed nutrient bottles and frozen for water column nutrient analysis. Approximately 0.7 L of the sample was dispensed into a 1 L screw capped polyethylene bottle and transported to the laboratory on ice under dark conditions for the quantification of suspended solids, particulate organic nitrogen (PON) and background levels of ^15^N in suspended particulates (phytoplankton and bacteria). Of the remainder of the water sample, approximately 1 L, was dispensed into a 1 L screw-capped polyethylene bottle and fixed with Lugol’s iodine for qualitative and quantitative plankton analysis. Pore water samples were also collected in triplicate using the air-stone based pore water sampler described in Nayar *et al*.^18^. The 60 mL syringe with which the pore water sample was collected was capped upon collection underwater, with the sample processed immediately on the boat. About 50 mL of this sample was filtered using a syringe filter (0.2 μm pore size) and transferred into labelled, pre-rinsed nutrient bottles and frozen for pore water nutrient analysis. Water quality parameters such as Photosynthetically Active Radiation (PAR), temperature, dissolved oxygen (DO), salinity, and pH were measured with sensors and dataloggers integrated with the benthic chambers. Salinity was measured using a multi-parameter water quality meter on site. Triplicate samples of *Zostera nigricaulis* were collected from each of the sites using a 24 cm diameter corer for the measurement of background levels of ^15^N in above-ground biomass (leaves and stalks), below-ground biomass (rhizomes and roots) and epiphytes. The cored seagrass samples were transported in mesh bags under dark conditions for processing in the laboratory.

### Nutrient uptake study

#### Description of benthic chambers used for nutrient uptake studies

**Figure 2:**
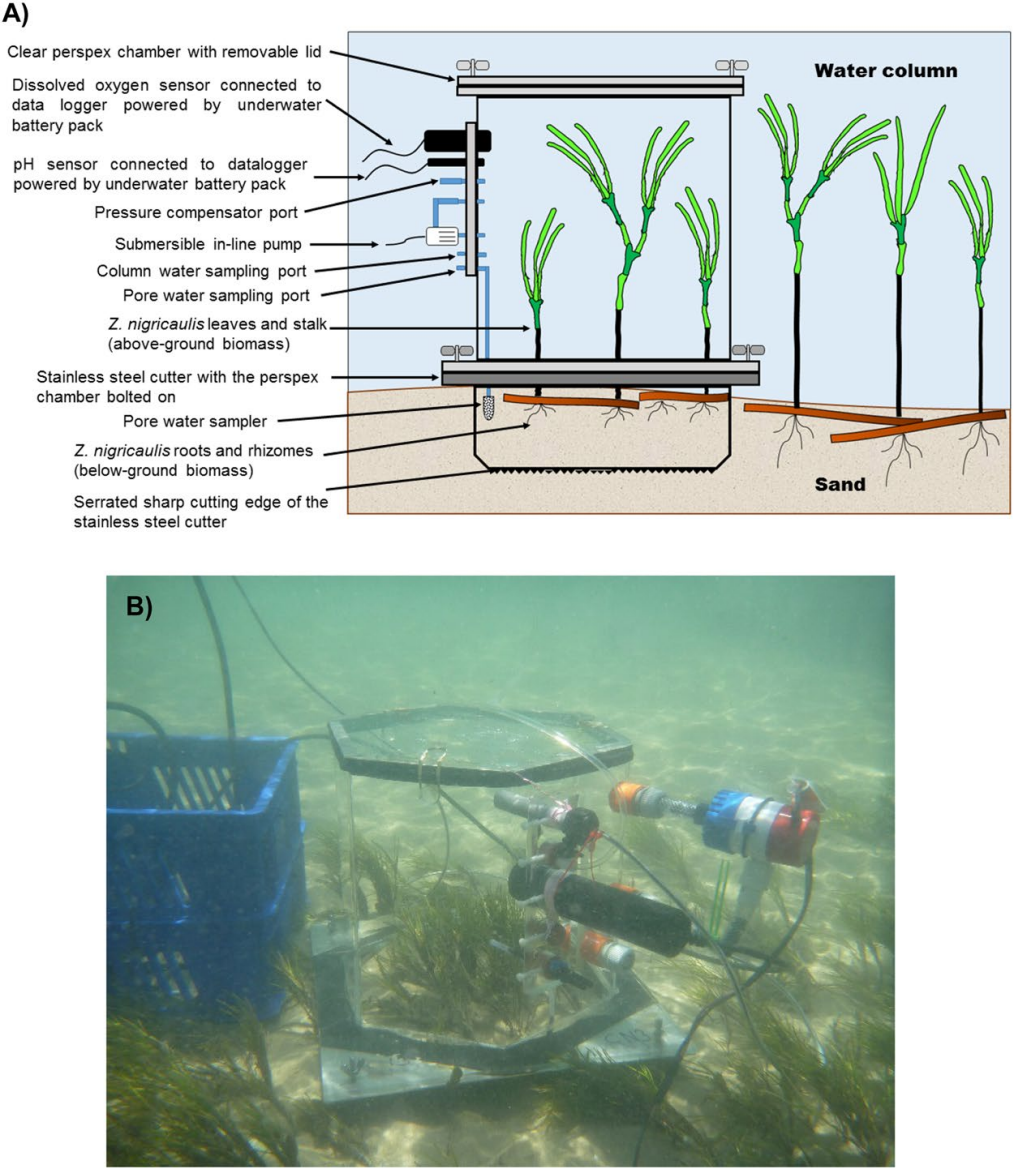
Benthic chambers used for uptake studies. (**A**) Schematic cross-sectional diagram of the chamber with its individual components, and (**B**) Close-up of a chamber over a *Zostera nigricaulis* bed with data logging probes, circulation pump, underwater power source and the pore water sampler.

Benthic chambers used in nutrient uptake studies comprised six identical cylindrical units made of clear perspex, each with an overall volume of 26 L (Fig. 2A,B). Each chamber had an inflow and an outflow connection onto which a pump line was connected to recirculate water contained within the chamber. The pump line consisted of a fibre reinforced PVC hose linked to the intake of a submersible in-line pump. The pumps were powered by a 12 V DC underwater battery pack that also powered a data logger to which dissolved oxygen and pH sensors were connected. The chambers were built with sampling ports for pore water and chamber water collections using a syringe. These sampling ports terminated with a two-way valve that isolated the chamber from the surrounding water. A pore water sampler made with an air stone diffuser was hooked to the pore water sampling port, using a tygon tube internally within the chamber. The stainless steel cutters to which the chambers were bolted had a sharp cutting edge with a square platform. Rubber washers were glued on to the platform to provide a tight seal between the chamber and the cutter after the chamber is bolted down. Each cutter had a volume of 8.45 L and covered an area of 0.0845 m^2^ when pushed into the sediment. A collapsible pressure compensation bag was connected to each of the six chambers to compensate for the reduction in the volume of water contained in the chamber as a result of water samples being drawn by syringes from the chamber. The bag provided pressure relief and prevented pore water from being upwelled into the chamber due to a reduction in pressure brought about by a reduction in volume. At the commencement of the trial, the pressure compensator bags were fully inflated.

#### Nutrient uptake experiments

Six stainless steel cutters were driven into a homogenous patch of *Zostera nigricaulis* beds by divers prior to the experiments. Care was taken to ensure minimal damage to seagrass while ensuring that the cutters were driven at least 10 cm into the sediment. The cutters also isolated the experimental plants from the surrounding plants so that any translocation of nutrients from outside the chambers would not occur.

The Perspex chambers were bolted on to the cutters on the seagrass patch ensuring that the leaves of the seagrass were carefully contained within the chambers whilst accomplishing a water tight seal between the cutter and the chamber. Three of these chambers were used for labelled ammonium uptake experiments, and three for labelled nitrate uptake experiments. The pump lines, pressure compensators and the sensors were hooked to the chambers. The pore water sampler was driven 2 cm into the sediments and the tygon tube from the sampler hooked to the sampling port of the chamber. Pumps were then hooked up and powered on to maintain water flow in the chambers.

Nutrient stock solutions (500 mg L^−1^) for spiking were prepared from labelled salts of ^15^NH_4_Cl (^15^N, 98%, Novachem Pty Ltd) and K^15^NO_3_ (^15^N, 99.22%, Novachem Pty Ltd) for ammonium and nitrate uptake trials, respectively. Nutrient spike solution was loaded into 10 mL syringes, sealed with an end cap. Each chamber was then spiked with the nutrient solution to yield a final concentration of 192 μg L^−1^ (13.7 μmol L^−1^) of the nutrient in each of the six chambers, simulating realistic elevated levels in the environment. Whilst the spike concentration is about an order of magnitude higher than the ambient nutrient concentrations measured in this study, it was based on higher levels reported historically from Port Phillip Bay (EPA^36^; A. Hirst; pers. comm.). The decision to use the higher spike concentration was also to ensure that the chambers were not totally depleted of nutrients at the end of the incubation. Approximately 60 mL of the column water and pore water were drawn using pre-labelled and end capped syringes to determine the initial concentrations of inorganic nutrients.

The chambers were then incubated for 2 hours. At the end of the incubation, about 120 mL and another 60 mL of water sample were drawn from each chamber using an end-capped syringe to measure uptake of nutrients by phytoplankton and bacteria (suspended particulates) and for the final concentrations of inorganic nutrients, respectively. Seagrass samples from each chamber were cored out in the manner described previously and transported to the laboratory in a mesh bag under dark conditions for biomass and nutrient uptake measurements.

### Nutrient translocation study

#### Description of benthic chambers used for translocation studies

**Figure 3:**
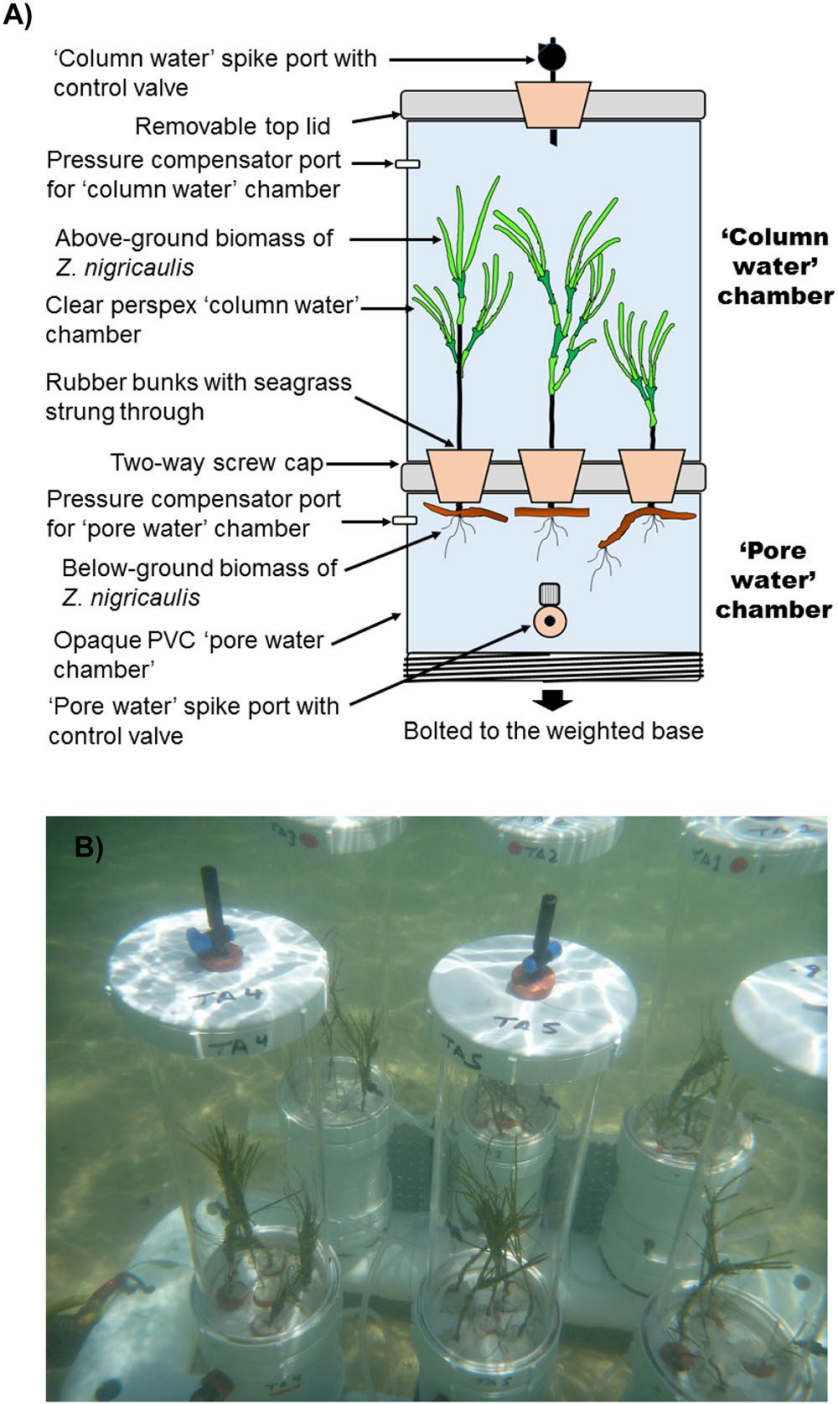
Benthic chambers used for translocation studies. (**A**) Schematic cross-sectional diagram of the translocation chamber with its individual components, and (**B**) Close up of the top chamber with the two-way screw cap. Individual plants are threaded through a rubber bunk in such a way that the above-ground biomass remain in the top clear Perspex chamber (column water) and the below-ground biomass remains in the chamber below (pore water).

The benthic chambers used for translocation studies comprised an upper chamber and a lower chamber (Fig. 3A,B). The upper chamber was made of clear Perspex tube with a lid on one end and a two-way screw cap on the other. The Perspex tube measured 29.5 cm in length and approximately 9.5 cm diameter with an overall volume of approximately 1.56 L. One end of the two-way screw cap screwed on to the upper perspex chamber, while the bottom end screwed onto the top part of the bottom chamber. The bottom chamber was made of opaque PVC pipe and measured 11.1 cm in length and 9.5 cm in diameter with a volume of approximately 0.78 L. The two-way screw cap also had five holes of 1 cm diameter each, drilled into it. This enabled the seagrass to be strung through in such a way that the top clear compartment held the above-ground biomass (simulating column water) and the opaque bottom chamber held the below-ground biomass (simulating pore water). Rubber bungs with slits were used to seal off the holes after the plants were strung through. Both the upper and bottom chambers were connected to a set of 60 mL syringes with tygon tubing as part of the pressure compensator assembly. Both the upper and bottom chambers were provided with sampling ports with a two-way valve to enable collection of water samples using a syringe. Six of these assembled chambers (upper and bottom) were bolted on to a base that was weighted down to the sea bed for stability during incubation.

#### Translocation experiments

Seagrass plants were uprooted carefully from the each bed ensuring their leaves, stalk, rhizomes and roots remained intact. Each plant was carefully threaded through the slit rubber bungs in such a way that the leaves and stalk remained above the bungs and the roots and rhizomes below. The rubber bungs were then secured into the pre-drilled holes on the two-way screw cap plate. Putty was used to ensure a good watertight seal between the top and bottom chamber. The Perspex chamber was screwed on to the two-way screw cap such that the above-ground biomass was contained within it. This whole assembly was screwed on to the bottom chamber already secured on the stand such that the below-ground biomass was contained within it. Like all other studies, this study is undertaken with the assumption that the seagrasses extracted from the sediments behave no different to those in the sediment.

Three chambers each had their column water (top chamber) spiked with labelled ammonium or nitrate, while a separate set of chambers had their pore water (bottom chamber) spiked with the labelled nutrients. The column (top chamber) was spiked to achieve a final concentration of 192 μg L^−1^ (13.7 μmol L^−1^) of ^15^N (^15^NH_4_ or ^15^NO_3_) and the pore water (bottom chamber) spiked to achieve 1602 μg L^−1^ (~114 μmol L^−1^) of ^15^N. Immediately upon spiking, water samples of about 0.05 L were collected from all the 12 upper and bottom chambers using pre-labelled and end-capped syringes for the initial concentrations of inorganic nutrients.

The chambers were then incubated for two hours. At the end of the incubation, another 60 mL of the water sample was drawn from each chamber using an end capped syringe to measure the final concentrations of inorganic nutrients (unutilised nutrients). Seagrass samples from each chamber were packed in labelled zip-lock bags, frozen and stored under dark for further processing.

### Laboratory analysis

All glassware used in the laboratory for processing samples were rinsed in AR grade methyl alcohol and then ‘baked’ in a furnace at 150 °C prior to use. All laboratory work-benches and equipment coming in contact with the samples were cleaned with AR grade methyl alcohol prior to use.

#### Background samples

Cored seagrass samples for biomass estimation was rinsed in clean, filtered seawater, and cleaned of drift algae, epibionts, dead leaves and sediments. Wet weight measurements of the above-ground biomass, below-ground biomass and epiphytic biomass were made. Moisture content of the sub-samples of the above- and below-ground biomass and epiphytes was assessed gravimetrically after freeze-drying the samples. Both, the above- and below-ground biomass were expressed on a dry weight basis. Epiphytes were scraped off the above-ground biomass. Epiphyte loading was deducted from the above-ground biomass to obtain the corrected above-ground biomass values on a dry weight basis; these values were then used for subsequent calculations.

For background levels of ^15^N in the phytoplankton, 0.15 L of the water sample was filtered through a GF/F filter paper (nominal pore size 0.4 μm) in triplicate for each site. The filter paper was folded and covered in aluminium foil and frozen at −40 °C until freeze-dried. Frozen samples were directly freeze-dried. The dried filter papers with suspended particulates were sent to the Isotope laboratory at Monash University for quantification of background levels of ^15^N.

PON was measured in triplicate for each site by filtering 0.5 L through a GF/F filter paper (nominal pore size 0.4 μm). Upon filtration, the filter papers with suspended particulates were stored in clean glass bottles at −40 °C until freeze-dried. Total suspended particulate concentration was measured gravimetrically adopting standard procedures^37^. The filter papers were then used for the analysis of PON by the alkaline persulphate digestion colorimetric procedure^38^. A Lachat Quickchem 8000 auto-analyser was used for colorimetric analysis.

Qualitative and quantitative analysis of plankton was carried out by concentrating 1 L of the Lugol fixed water sample to 25 mL using a separating flask. From the concentrated sample, a 1 mL aliquot was pipetted onto a Sedgewick-Rafter cell. A light microscope was used for identification and cell counts. The abundance of phytoplankton was expressed as the number of cells per litre (cells L^−1^).

#### Nutrient uptake experiments

When processing biological samples from nutrient uptake experiments great caution was exercised to ensure no cross contamination of the samples. Epiphytes were carefully scraped off the seagrass leaves (10 leaves) and stalk using a clean scalpel. Scraped epiphytes were collected and transferred into a clean glass scintillation vial. The scraped seagrass leaves and stalk were weighed and their length and width recorded. The above-ground biomass was then transferred into a clean glass bottle. Likewise, the below-ground biomass, comprising both rhizome and roots, was weighed and stored in a clean glass bottle. About 0.12 L of the enriched water samples was collected from the spiked benthic chambers for the quantification of phytoplankton and phytoplankton uptake rates. Samples for phytoplankton uptake rates were filtered through a GF/F filter paper (nominal pore size 0.4 μm) under vacuum. The filter papers with suspended particulates were transferred into clean bottles for subsequent processing, storage and analysis. Since it was impossible to segregate bacterial uptake from phytoplankton uptake, what is described in this study as plankton uptake is in fact a combined uptake by phytoplankton and bacteria. Because of the likelihood of high spatial variability associated with plankton distribution, plankton measurements in this study were made at each of the 3 study sites for mass balance budgets. The filter papers with suspended particulates were stored in the dark at −40 °C. Upon thawing, the samples were immediately freeze-dried. Dry-weight of epiphytes was recorded to calculate epiphyte loading, and expressed as dry-weight biomass per unit dry weight and unit area of seagrass leaves and stalk.

To measure background levels of plant tissue nutrients and uptake of ^15^N labelled nitrogen from the water column by above- and belowground biomass of seagrass and epiphytes, freeze-dried samples were pulverised in a ball mill grinder. A sub-sample of the pulverised material was analysed at the Stable Isotope Laboratory, Research School of Biology, Australian National University for the determination of nitrogen content (mg) and atom % ^15^N in the tissues. Isotopic and elemental analyses of nitrogen were performed on a continuous-flow stable isotope ratio mass spectrometer. Aliquots of about 4 to 5 mg of dried and pulverised samples were weighed into tin foil cups and loaded into an elemental analyser used as a preparative system for the mass spectrometer. Samples were combusted oxidatively and the resulting gases dried before separation in a column operating at room temperature. The CO_2_ peak was typically diluted to be about 10 times the size of the N_2_ peak, but both absolute and relative sizes were highly variable due to the nitrogen content of the seagrasses and perhaps the amount of inert material mixed with the seagrass compounds. To measure background levels and enriched phytoplankton, the filter papers with suspended particulates were analysed at the Water Studies Centre, Monash University in an elemental analyser interfaced to a continuous-flow isotope ratio mass-spectrometer.

Specific uptake rates of various components were calculated with assumptions outlined by Cornelisen and Thomas^39^ using equations from Nayar *et al*.^18^. Specific uptake rates for each component, when multiplied with their respective biomass yielded the total uptake rate for that component in a chamber. The sum of uptake rates for each component in the chamber is referred to as the ‘total component uptake’ in the chamber. Total component uptake reflected the greater contribution of the overall uptake of a component whose biomass was greater. The component here refers to above-ground biomass (leaves and stalks), below-ground biomass (rhizomes and roots) or epiphytes. These values for total component uptake and the total input were used to estimate the percentage of nutrient resource allocated to each component^18^.

#### Translocation experiments

For each chamber the above-ground biomass was separated from the below-ground biomass. The seagrass tissues were processed for ^15^N enrichment as described above. Translocation rate of labelled ammonium and nitrate were calculated from the net ^15^N gain in the opposite compartment to that spiked. Percentage uptake and translocation were calculated from the total ^15^N assimilated by the two compartments. The sum of uptake and translocation is referred to as the total nitrogen resource assimilated by the seagrass. The total resource assimilated is standardised to 100%.

### Statistical analysis

Differences in uptake rates were analysed using a two-way permutation-based analysis of variance (PERMANOVA) with 4 components by 2 nitrogen sources as fixed factors. The four components were seagrass leaf and stalk (above-ground), seagrass roots and rhizomes (below-ground), epiphytes and plankton with the two nitrogen sources being ammonium and nitrate. The analyses were based on Euclidean distances with P-values calculated from 9,999 permutations of the residuals under a reduced model using the PRIMER 6.1.6 software package^40^ and PERMANOVA+^41^. When required, post hoc pairwise tests were used to determine which components differed.

Data for translocation and uptake of ammonium and nitrate by above-ground (leaf) and below-ground (roots and rhizomes) compartments were analysed by Analysis of Variance (ANOVA). *Post hoc* multiple comparison were carried out using Tukey’s test to ascertain significant differences, if any, between translocation and uptake by above-ground and below-ground compartments for ammonium and nitrate for the three study sites. The statistical software Minitab Ver. 17.1.0 was used for analysis.

All statistical comparisons were considered significant at P < 0.05.

## Results

### Environmental characteristics of the chamber deployment sites

Background physical, chemical and biological parameters were measured at the three study sites where the benthic chambers were deployed. Mean background levels of the various physico-chemical and biological parameters monitored are summarised in Table 1.

**Table 1:** Summary of background physical, chemical and biological data collected at Blairgowrie, Swan Bay and Kirk Point in Port Phillip Bay during chamber deployment.

Parameters	n	Blairgowrie	Swan Bay	Kirk Point
Photosynthetically Active Radiation (μmol photons m^−2^ s^−1^)	228	832.3 ± 260.9	ND	ND
Total Suspended Solids (mg L^−1^)	3	60.8 ± 12.8	58.3 ± 3.7	55.8 ± 3.6
Water temperature (°C)	352	20.0 ± 0.6	21.8 ± 0.2	21.7 ± 0.3
pH in chamber	352	9.4 ± 0.0	10.0 ± 0.1	9.3 ± 0.0
Dissolved oxygen in chamber (mg L^−1^)	352	10.05 ± 0.11	9.07 ± 0.84	11.02 ± 0.64
Salinity (‰)	3	36.9 ± 1.27	39.3 ± 0.49	35.4 ± 0.44
Column water dissolved NH_3_ (μg L^−1^)	3	8	25 ± 3	7 ± 1
Pore water dissolved NH_3_ (μg L^−1^)	3	75 ± 50	367 ± 329	167 ± 50
Column water dissolved NO_x_ (μg L^−1^)	3	13	10 ± 0	9 ± 1
Pore water dissolved NO_x_ (μg L^−1^)	3	13 ± 2	112 ± 70	32 ± 21
Column water dissolved PO_4_ (μg L^−1^)	3	37	10 ± 0	111 ± 16
Pore water dissolved PO_4_ (μg L^−1^)	3	73 ± 9	143 ± 12	211 ± 131
Epiphyte loading (g DW. g^−1^ DW)	3	0.311 ± 0.14	0.308 ± 0.04	0.131 ± 0.06
Epiphyte loading (g DW cm^−2^)	3	2.225 ± 0.42	1.445 ± 0.4	0.317 ± 0.02
Above-ground seagrass biomass cover (g DW m^−2^)	3	141.6 ± 7.18	142.6 ± 7.65	41.7 ± 16.48
Below-ground seagrass biomass cover (g DW m^−2^)	3	77.2 ± 56.76	134.0 ± 18.3	59.5 ± 19.13
Phytoplankton counts (cells L^−1^)	3	842 ± 165	835 ± 261	905 ± 438
Above-ground seagrass biomass nitrogen (mg N g^−1^)	3	11.33 ± 1.15	8.8 ± 3.25	9.97 ± 0.06
Below-ground seagrass biomass nitrogen (mg N g^−1^)	3	7.93 ± 1.96	5.73 ± 0.49	6.43 ± 0.35
Epiphyte nitrogen (mg N g^−1^)	3	28.84 ± 2.06	22.66 ± 2.69	10.46 ± 3.86
Particulate nitrogen (mg N g^−1^)	3	1.84 ± 0.32	2.67 ± 0.13	11.66 ± 3.71
Above-ground seagrass biomass δ^15^N enrichment (‰)	3	9.43 ± 0.42	4.80 ± 0.20	21.35 ± 3.70
Below-ground seagrass biomass δ^15^N enrichment (‰)	3	18.62 ± 5.49	5.87 ± 0	17.69 ± 4.49
Epiphytes δ^15^N enrichment (‰)	3	7.34 ± 0.59	5.34 ± 0.85	15.86 ± 0.26
Plankton δ^15^N enrichment (‰)	3	10.30 ± 0.28	2.45 ± 0.64	21.00 ± 0.30

All values are means ± standard deviation. The abbreviation ‘ND’ represents ‘No data’.

Measurements for Photosynthetically Active Radiation (PAR) could only be undertaken at Blairgowrie, as the data logger failed on deployments at Swan Bay and Kirk Point. Visual observations of the light conditions at the three sites on the three days of deployment were consistent. Mean PAR values at Blairgowrie were 832.3 ± 260.9 μmol photons m^−2^ s^−1^. Total Suspended Solids (TSS) concentrations ranged between 58 and 61 mg L^−1^. TSS concentrations across the study sites were consistent. Water temperature was also consistent during the course of the deployment and across the three sites, ranging from 20.0 to 21.8 °C.

Measurement of pH within the chamber revealed stability in pH over the duration of the deployment. Marginally higher pH values were recorded at Swan Bay (10.0 ± 0.1) in contrast to Blairgowrie (9.4) and Kirk Point (9.3). High pH values within the chamber are attributed to seagrass photosynthesis. Dissolved oxygen (DO) concentrations within the chamber varied between 9.07 to 11.02 mg L^−1^ at the three sites, with highest concentrations at Kirk Point and lowest at Swan Bay. Ambient salinity varied between 35.4 ppt at Kirk Point and 39.3 ppt at Swan Bay.

Among the nutrients, ammonium concentrations in the water column were higher at Swan Bay compared to Blairgowrie or Kirk Point. None of the sites stood out from any other with regards to the pore water ammonium concentrations. Overall, ammonium concentrations ranged from 7 to 25 μg L^−1^ in the water column and 75 to 367 μg L^−1^ in the pore water. Oxidised nitrogen concentrations (NO_x_) in the water column ranged between 9 and 13 μg L^−1^, with the highest concentrations recorded at Blairgowrie followed by Swan Bay and Kirk Point. Swan Bay had higher pore water oxidised nitrogen concentrations than Kirk Point or Blairgowrie. Concentrations ranged between 13 to 112 μg L^−1^. Dissolved phosphate concentrations were highest at Kirk Point, both in the column water and pore water. Phosphate concentrations ranged between 10 and 111 and 73 and 211 μg L^−1^ respectively, in the column water and pore water across the three sites.

Epiphytic loading in relation to the dry weight of the leaf tissue or leaf area did not differ between Blairgowrie and Swan Bay. The amount of epiphytes growing on seagrass at Kirk Point was much lower than the other two study sites. Overall, epiphytic loading ranged between 0.13 and 0.31 g DW g^−1^ DW and between 0.32 and 2.23 g DW cm^−2^. Epiphyte tissue nitrogen concentrations were higher than seagrass tissue, ranging between 10.46 and 28.84 mg N g^−1^ DW at Kirk Point and Blairgowrie. Spatially, nitrogen content in epiphytic tissue was comparable between Blairgowrie and Swan Bay, with much lower concentrations at Kirk Point.

Above-ground seagrass biomass was similar at Blairgowrie and Swan Bay, but lower at Kirk Point, and ranged between 41.7 and 142.6 g DW m^−2^. The below-ground seagrass biomass was higher at Swan Bay than Blairgowrie or Kirk Point. The below-ground biomass was very variable between samples collected at Blairgowrie. The below-ground biomass ranged between 59 and 134 g DW m^−2^ across the three sites.

Mean phytoplankton cell counts ranged from 842 to 903 cells L^−1^. No distinct spatial variation was observed in phytoplankton abundance. The phytoplankton community was dominated by diatoms ranging in abundance from 85% (of the total abundance) at Swan Bay to 93% at Blairgowrie (Table 2). Cyanophytes were only recorded at Swan Bay (15%) and Kirk Point (2%). Dinoflagellates were observed at Kirk Point (12%) and Blairgowrie (6%), whilst, chlorophyceae was only recorded at Blairgowrie (2%).

**Table 2:** Qualitative and quantitative distribution of phytoplankton (cells L^−1^) at Blairgowrie, Swan Bay and Kirk Point in Port Phillip Bay during chamber deployment.

Species	Blairgowrie		Swan Bay		Kirk Point	
	Mean	SD	Mean	SD	Mean	SD
**Chlorophyceae**						
Miscellaneous Chlorophytes	16	27	—	—	—	—
**Bacillariophyceae**						
*Amphora* sp.	47	81	15	27	16	27
*Asterionellopsis glacialis*	16	27	—	—	—	—
*Bacillaria paradoxa*	—	—	31	27	—	—
*Bellerochea* sp.	16	27	—	—	—	—
*Chaetoceros* sp.	—	—	—	—	62	27
*Cocconeis* sp.	—	—	31	27	—	—
*Coscinodiscus* sp.	—	—	16	27	—	—
*Cyclotella* sp.	—	—	31	53	—	—
*Dactyliosolen* sp.	—	—	31	53	—	—
*Eucampia* sp.	16	27	—	—	—	—
*Grammatophora* sp.	—	—	—	—	31	54
*Guinardia* sp.	109	188	—	—	—	—
*Guinardia striata*	31	54	—	—	—	—
*Gyrosigma* sp.	16	27	—	—	—	—
*Leptocylindrus danicus*	—	—	15	27	—	—
*Leptocylindrus* sp.	16	27	—	—	—	—
*Licmophora* sp.	—	—	15	27	—	—
*Navicula* sp.	78	27	93	47	62	72
*Nitzschia* sp.	140	140	62	27	188	94
*Paralia sulcata*	47	47	—	—	—	—
*Pleurosigma* sp.	—	—	77	71	47	47
*Pseudo-nitzschia* sp.	31	54	15	27	—	—
*Rhizosolenia* sp.	—	—	—	—	31	54
*Skeletonema* sp.	—	—	—	—	94	124
*Thalassionema nitzschioides*	—	—	31	27	16	27
*Thalassionema* sp.	62	28	15	27	78	136
*Thalassiosira* sp.	—	—	15	27	16	27
Miscellaneous diatoms	156	98	217	176	141	47
**Cyanophyceae**						
*Chamaesiphon* sp.	—	—	—	—	16	27
*Oscillatoria* sp.	—	—	124	97	—	—
**Dinophyceae**						
*Pyrophacus* sp.	31	54	—	—	—	—
Miscellaneous dinoflagellates	16	27	—	—	109	188

All values are mean ± standard deviation with n = 3.

Nitrogen content in the above-ground biomass of seagrass ranged from 8.8 to 11.33 mg N g^−1^ DW across the study area with no variation between the study sites. Similarly, the tissue nitrogen concentrations in the below-ground biomass varied between 5.73 and 7.93 mg N g^−1^ DW, with no variation between the three sites. Particulate nitrogen concentrations were distinctly different between the sites, with Kirk Point registering the highest concentrations, followed by Swan Bay and then Blairgowrie. These concentrations ranged from 1.84 to 11.66 mg N g^−1^ DW.

Similar to tissue nitrogen concentrations, δ^15^N enrichment in above-ground seagrass biomass registered distinct spatial patterns with the highest enrichment observed at Kirk Point followed by Blairgowrie and lowest at Swan Bay. Enrichment in above-ground seagrass biomass ranged from 4.80 to 21.35‰. A comparable pattern was also observed with epiphytes, where the enrichment ranged from 5.34 to 15.86‰. With the below-ground seagrass tissue however, highest enrichment was observed at Blairgowrie and Kirk Point, with the lowest levels recorded from Swan Bay. Enrichment ranged from 5.87 to 18.62‰. In the case of phytoplankton, the trend was comparable to the enrichment in above-ground seagrass biomass, with the highest enrichment at Kirk Point followed by Blairgowrie and lowest at Swan Bay. Enrichment in phytoplankton ranged from 2.45 to 21.00‰.

### Quantification of nutrient uptake rates

**Figure 4:**
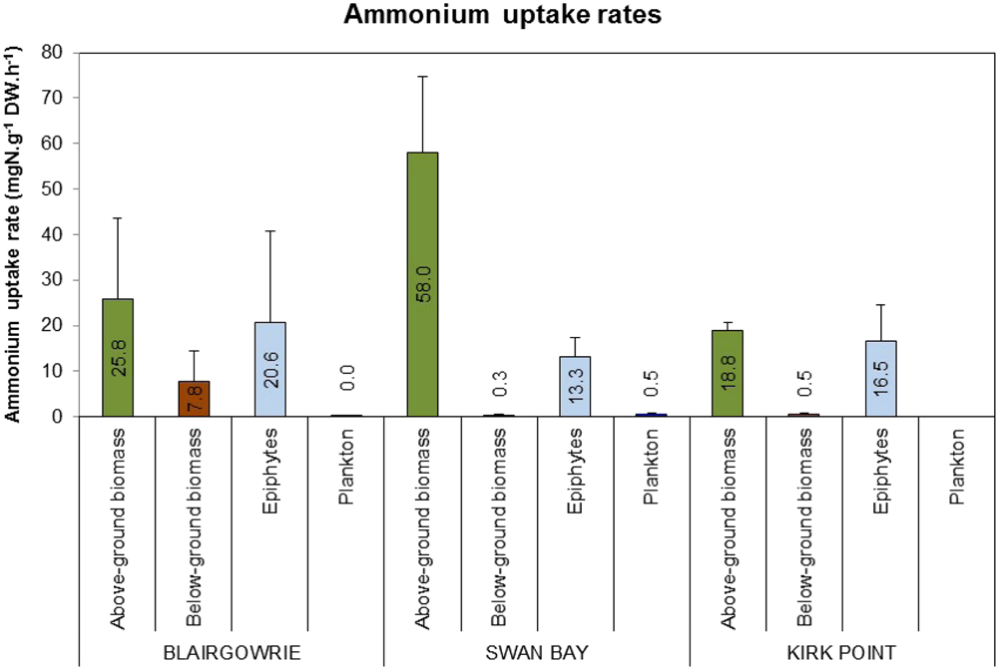
Mean uptake rates of ammonium (μg N g^−1^ DW h^−1^) by above-ground seagrass biomass, below-ground seagrass biomass, epiphytes and plankton in *Zostera nigricaulis* beds at Blairgowrie, Swan Bay and Kirk Point in Port Phillip Bay. The error bars depict standard deviation (n = 3). Note there are no data for plankton uptake at Kirk Point.

Uptake of ammonium by above-ground seagrass biomass was highest (18.8–58.0 μg N g^−1^ DW h^−1^) followed by epiphytes and lowest by the below-ground seagrass biomass (0.3–7.8 μg N g^−1^ DW h^−1^). Uptake rates for epiphytes and plankton were 13.3–20.6 μg N g^−1^ DW h^−1^ and 0.05–0.53 μg N g^−1^ DW h^−1^, respectively. Site-specific variations were observed with higher uptake rates observed at Swan Bay (Fig. 4).

**Figure 5:**
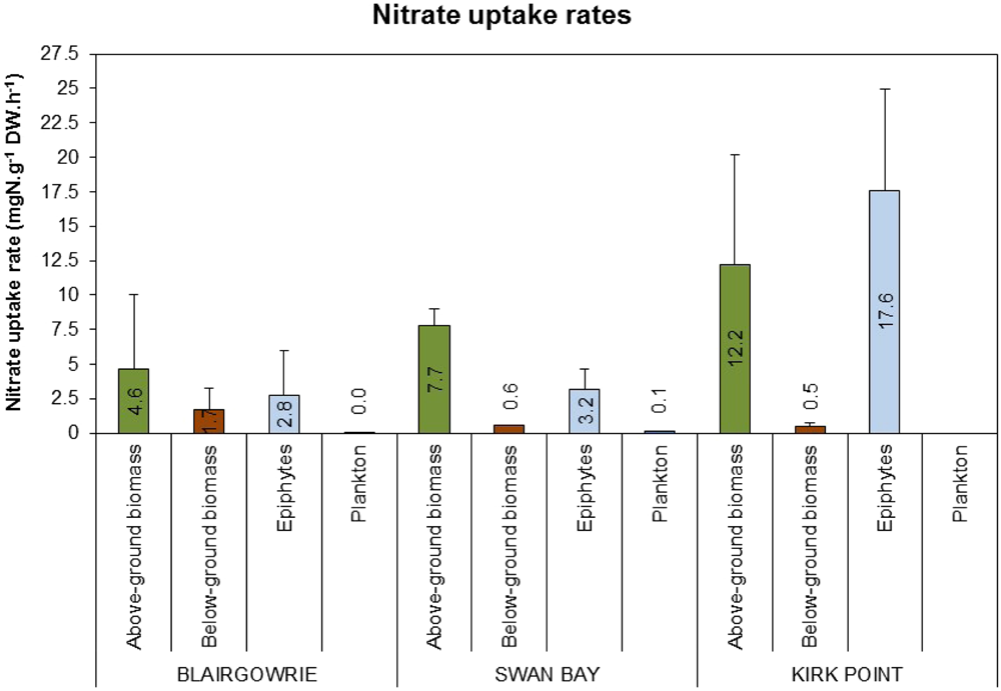
Mean uptake rates of nitrate (μg N g^−1^ DW h^−1^) by above-ground seagrass biomass, below-ground seagrass biomass, epiphytes and plankton in *Zostera nigricaulis* beds at Blairgowrie, Swan Bay and Kirk Point in Port Phillip Bay. The error bars depict standard deviation (n = 3). Note there are no data for plankton uptake at Kirk Point.

Uptake of nitrate was highest at Kirk Point followed by Swan Bay and then Blairgowrie. As with ammonium, above-ground seagrass biomass dominated the rate of uptake of nitrate (4.6–12.2 μg N g^−1^ DW h^−1^) followed by epiphytes (2.8–17.6 μg N g^−1^ DW h^−1^), roots (0.5–1.7 μg N g^−1^ DW h^−1^) and lowest by plankton (0.01–0.09 μg N g^−1^ DW h^−1^; Fig. 5).

At Blairgowrie, uptake of ammonium by biotic components was significantly higher than the uptake of nitrate, but there were no differences between various biotic components (Table 3).

**Table-3:** Summarised results of PERMANOVA for uptake rates of ammonium and nitrate by biotic components (above-ground seagrass biomass, below-ground seagrass biomass, epiphytes and plankton) at each of the three study sites in Port Phillip Bay and their interactions. Figures in bold are significant at P < 0.05.

Site	Source	Degrees of freedom	Mean Sum of Squares	F	P
Blairgowrie	Components	3	274.32	2.5424	0.0916
	Nutrients	1	718.00	6.6546	0.0196
	Components × Nutrients	3	143.56	1.3305	0.3072
Swan Bay	Components	3	1424.7	30.189	0.0002
	Nutrients	1	936.64	19.846	0.0010
	Components × Nutrients	3	867.52	18.382	0.0001*
Kirk Point	Components	2	446.84	18.151	0.0010
	Nutrients	1	0.7115	0.0029	0.8648
	Components × Nutrients	2	72.325	2.9379	0.0895

*Significant interaction term.

Significant interactions were observed between the biotic components and the nutrient types at Swan Bay (Table 3). *Post hoc* analysis indicated that the uptake of ammonium by the above-ground seagrass biomass was significantly higher than the uptake by epiphytes, below-ground seagrass biomass and plankton. With nitrate on the other hand, uptake by the above-ground biomass did not differ from epiphytes, but did from below-ground biomass and plankton (Table 4). Uptake of nitrate by epiphytes was significantly higher than by the below-ground biomass and plankton. Similarly, uptake of nitrate by the below-ground seagrass biomass was significantly higher than plankton (Table 4). Leaf uptake of ammonium was significantly higher than nitrate (P = 0.0066). This was in contrast to the below-ground seagrass biomass, epiphytes and plankton, all of which showed no significant differences between the uptake of ammonium and nitrate (P > 0.05).

**Table 4:** Results of the *post hoc* pair-wise comparisons of uptake of ammonium and nitrate by various biotic components at the three sites in Port Phillip Bay.

Sites		*Post hoc* comparison
Blairgowrie		$\overline{{\rm{Above}}-{\rm{ground}}\,{\rm{biomass}} > {\rm{Epiphytes}} > {\rm{Below}}-{\rm{ground}}\,{\rm{biomass}} > {\rm{Plankton}}}$
Swan Bay	Ammonium	${\rm{Above}}-{\rm{ground}}\,{\rm{biomass}} > \overline{{\rm{Epiphytes}} > {\rm{Below}}-{\rm{ground}}\,{\rm{biomass}} > {\rm{Plankton}}}$
	Nitrate	$\overline{{\rm{Above}}-{\rm{ground}}\,{\rm{biomass}} > {\rm{Epiphytes}}} > {\rm{Below}}-{\rm{ground}}\,{\rm{biomass}} > {\rm{Plankton}}$
Kirk Point		$\overline{{\rm{Epiphytes}} > {\rm{Above}}-{\rm{ground}}\,{\rm{biomass}}} > {\rm{Root}}$

The biotic components are arranged in ascending order of their means with pairs not significantly different from each other linked by a continuous line (P < 0.05).

At Kirk Point nutrient uptake by the various biotic components varied significantly, with no significant differences between the uptake of ammonium and nitrate (Table 3). Pair-wise comparison of the biotic components showed significantly lower uptake rates by roots when compared to epiphytes and above-ground seagrass biomass (Table 4). No significant difference were observed in the uptake rates of above-ground seagrass biomass and epiphytes (P > 0.05).

### Resource allocation

**Figure 6:**
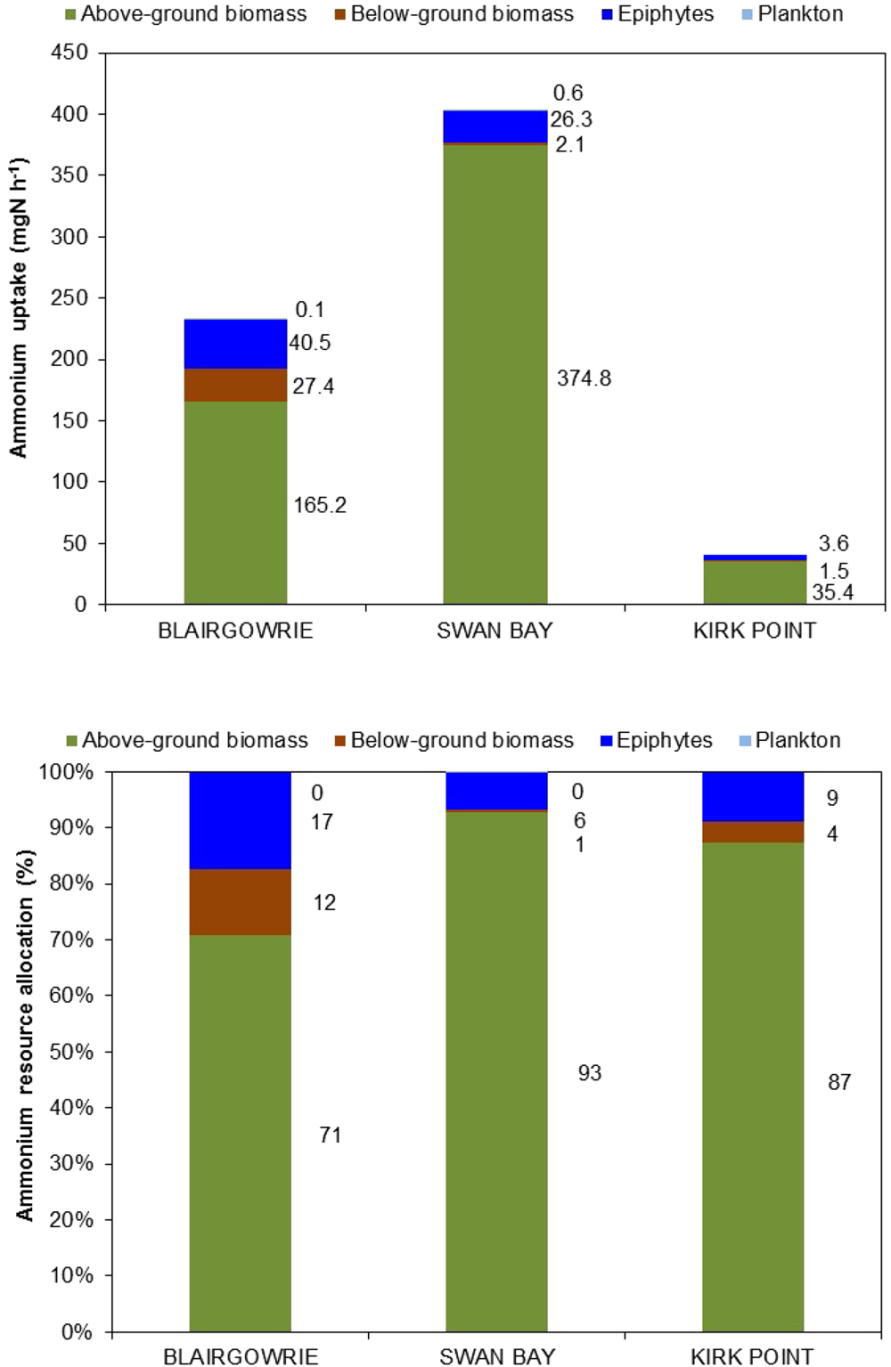
Total component uptake and resource allocation of ammonium by above-ground seagrass biomass, below-ground seagrass biomass, epiphytes and plankton in *Zostera nigricaulis* beds at Blairgowrie, Swan Bay and Kirk Point in Port Phillip Bay. The total component uptake accounts for the effect of differences in biomass for each of the biotic components. Note that the data for plankton are not included for Kirk Point.

Estimates of total component uptake of ammonium were highest at Swan Bay followed by Blairgowrie and Kirk Point (Fig. 6). Above-ground seagrass tissue accounted for between 71 and 93% of the total ammonium resource, followed by epiphytes (9–17%) and then below-ground seagrass biomass (1–12%). Plankton assimilation was observed to be negligible. Although the rate of assimilation of ammonium varied across the study sites, the pattern of resource allocation was fairly consistent across the study area.

**Figure 7:**
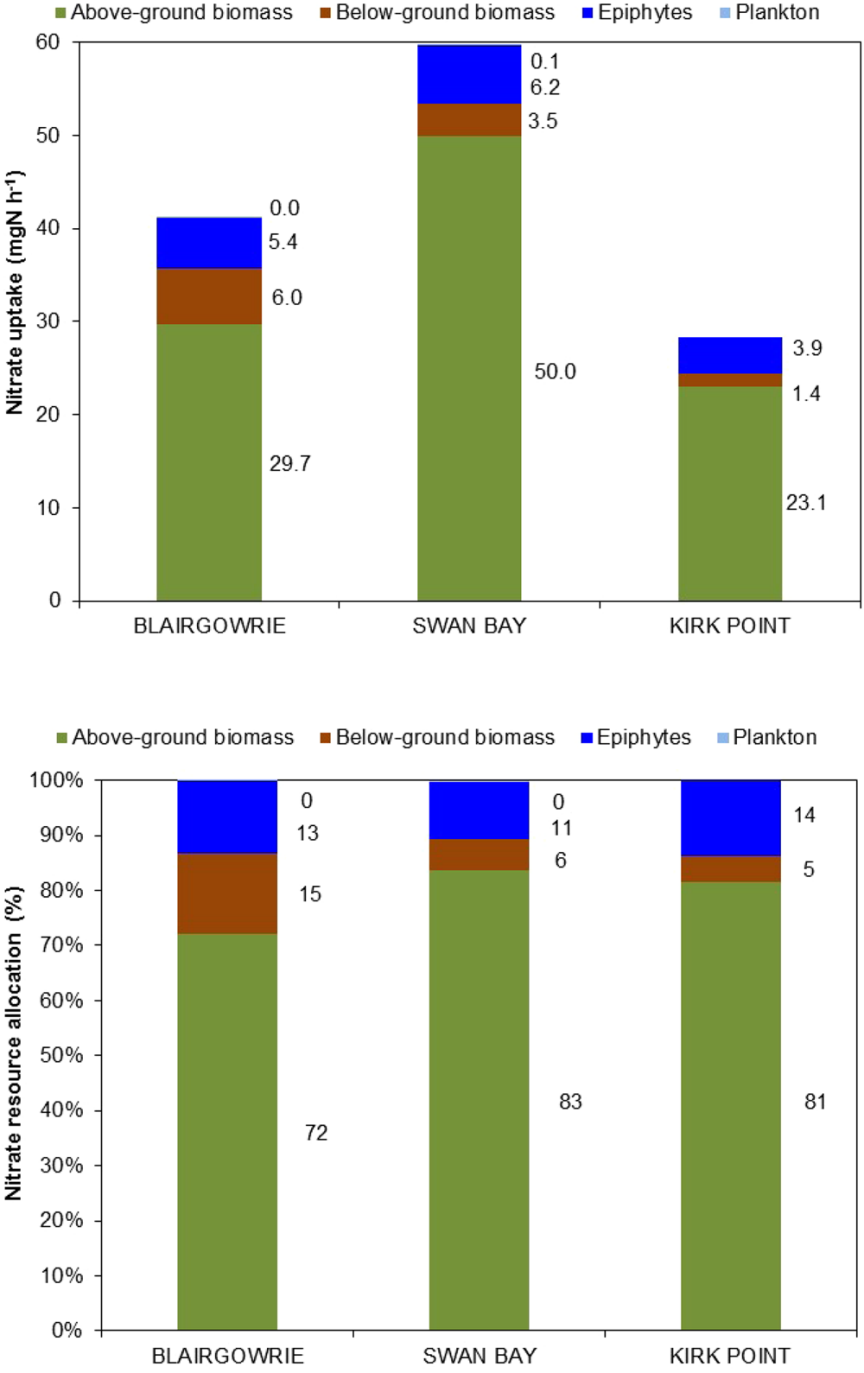
Total component uptake and resource allocation of nitrate by above-ground seagrass biomass, below-ground seagrass biomass, epiphytes and plankton in *Zostera nigricaulis* beds at Blairgowrie, Swan Bay and Kirk Point in Port Phillip Bay. The total component uptake accounts for the effect of differences in biomass for each of the biotic components. Note that the data for plankton are not included for Kirk Point.

The total component uptake of nitrate showed a similar pattern to ammonium, although the rate of nitrate assimilation was about one sixth of the ammonium assimilation, pointing to a preference for ammonium as a nitrogen source over nitrate (Fig. 7). Above-ground seagrass tissue dominated the assimilation of nitrate accounting for 72–83% of the total resource in contrast to epiphytes (11–13%) and below-ground biomass (5–15%). As with ammonium, assimilation of nitrate by plankton was negligible.

### Translocation rates and uptake

**Figure 8:**
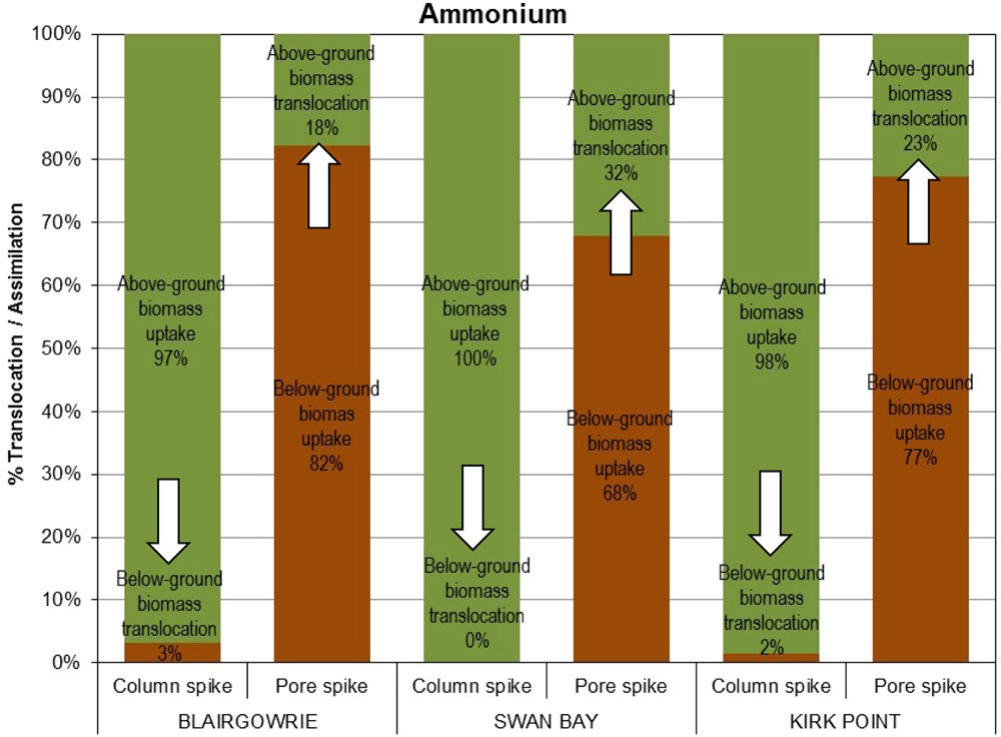
Translocation and uptake of ammonium by above-ground and below-ground biomass of *Zostera nigricaulis* at Blairgowrie, Swan Bay and Kirk Point in Port Phillip Bay. Percentage translocation is the amount of ammonium translocated to the above-ground or below-ground biomass, calculated as a percentage of the total ammonium assimilated by the seagrass. The arrows in the figure depict the movement of ammonium from one compartment to the other.

Overall, the uptake of ammonium by above-ground seagrass biomass accounted for a greater proportion of assimilation than the uptake by the below-ground biomass (Fig. 8). However, in the absence of any water column addition of ammonium, the below-ground seagrass biomass took up ammonium (68–82%) from the ‘pore water’ and translocated a fraction of the nitrogen pool to the above-ground seagrass biomass. Similarly, when enrichment of the water column occurred with no inputs into the pore water, the above-ground biomass took up ammonium (97–100%) and translocated a fraction of the nitrogen pool to the below-ground biomass. The fraction of the ammonium pool translocated from the below-ground biomass to the above-ground biomass (18–32%; acropetal translocation) was higher than the fraction translocated from the above-ground biomass to the below-ground biomass (0–3%; basipetal translocation) at all three sites. Translocation and uptake between the sites were not significantly different (ANOVA: *F*_2,33_ = 0.00, P = 1.000). However, assimilation and translocation by leaf and root compartments were different (ANOVA: *F*_3,32_ = 364.44, P < 0.001).

**Figure 9:**
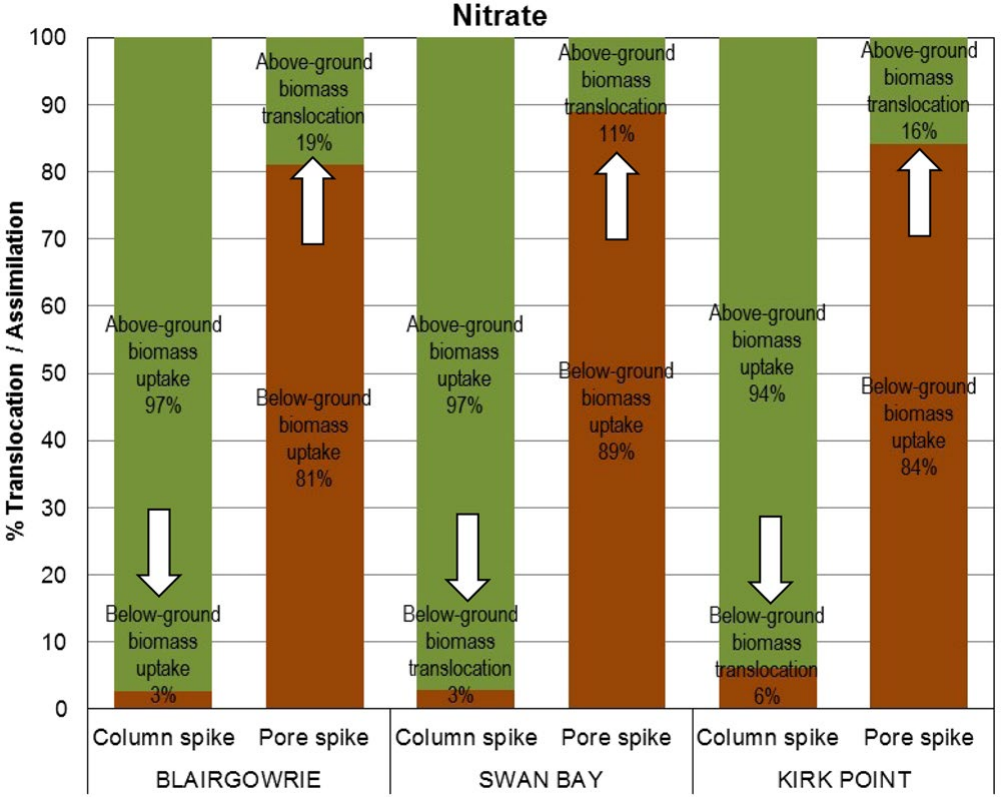
Translocation and uptake of nitrate by above-ground and below-ground biomass of *Zostera nigricaulis* at Blairgowrie, Swan Bay and Kirk Point in Port Phillip Bay. Percentage translocation is the amount of nitrate translocated to the above-ground or below-ground biomass, calculated as a percentage of the total nitrate assimilated by the seagrass. The arrows in the figure depict the movement of ammonium from one compartment to the other.

Translocation of nitrate exhibited a similar trend as ammonium, where the above-ground biomass assimilated a greater proportion of nitrate (94–97%), than the below-ground biomass (81–89%; Fig. 9). It was also observed that the pool of nitrate translocated from the below-ground biomass to the above-ground biomass (11–19%) was higher than the translocation of the nitrate pool from the above-ground biomass to the below-ground biomass (3–6%). Statistically, no significant differences were observed for translocation and uptake of nitrate between the sites (ANOVA: *F*_2,24_ = 0.00, P = 1.000). As with ammonium, the assimilation and translocation of nitrate by the above-ground and below-ground seagrass compartments were significantly different (ANOVA: *F*_3,24_ = 653.00, P < 0.001).

## Discussion

Like all terrestrial and aquatic plants, seagrasses require nitrogen to maintain their metabolic processes and productivity. In addition to recycling of nitrogen from internal nitrogen pools^42–44^, seagrasses are very dependent on their external media such as sediments and the water column to meet their nitrogen demand^13,23,26^. Port Phillip Bay, where this study was carried out, has been impacted by nitrogen inputs from the Waste Water Treatment Plant (WTP) since 1890, and runoff from the Yarra River since the 1840s. However, with improvements to the WTP and catchment management, the total nitrogen loads to PPB have declined since the 1990s. These improvements, coupled with natural processes such as denitrification, have considerably reduced the amount of nitrogen in the system, to the point that nitrogen is now regarded as a limiting nutrient^45^. Tissue nitrogen levels recorded from background samples in this study, being below the threshold of 1.8% (18 mg g^−1^ DW), are a possible indication of nutrient limitation^46^.

In the natural environment, nitrogen is available to seagrasses as a mixture of different sources that may include dissolved inorganic nitrogen species (such as ammonium and nitrate) as well as dissolved organic nitrogen species (such as urea and amino acids). Whilst some studies have reported acquisition of organic nitrogen sources by seagrasses^47–49^, especially at low ambient nitrogen concentrations, a large body of literature from various geographical regions points towards a preferential acquisition of inorganic nitrogen^13,18,27,50,51^. Among the two inorganic sources of nitrogen, the preferential uptake of ammonium over nitrate observed in this study complements the reported increased affinity for ammonium over nitrate by other seagrass species^13,18,21,25,52^. The preferential uptake of the reduced form of inorganic nitrogen (ammonium) by seagrasses has been attributed to physiological demands associated with the uptake of nitrate^13,53,54^. The assimilation of nitrate, the oxidised form of nitrogen, is energetically expensive as it involves an active transport system^55,56^. Under nutritionally poor (oligotrophic) conditions, seagrasses, in particular *Zostera*, have been reported to take up nutrients in whatever form is available from the water column or pore water^49,57^. This agrees with the findings of Alexandre *et al*.^27^ that ammonium was the preferential inorganic nitrogen source for *Zostera noltii*, with the affinity for nitrate increasing in the absence of ammonium. However, the authors suggested that in the presence of both nitrogen sources, ammonium was preferentially taken up over nitrate. This is also reflected in the specific uptake rates, where the uptake of ammonium in this study was about 6 times that of nitrate. Alexandre *et al*.^58^ similarly observed ammonium uptake rates to be on an average 10 times higher than nitrate uptake rates. In another study on *Zostera noltii*, the same authors reported a 30 fold increased affinity for ammonium compared with nitrate^27^. This affinity for ammonium by the leaves of *Zostera* has been stated to make this species better adapted to thrive under pulses of ammonium released from sediments to the water column with tidal changes experienced in intertidal zones^59^. As with seagrasses, the preferential uptake of ammonium over nitrate was also observed with other biotic components associated with the seagrass bed viz., phytoplankton and epiphytes. This clearly demonstrates that epiphytes and phytoplankton, like seagrasses, possess a greater affinity for ammonium as a nitrogen source than for nitrate.

Specific uptake rates of ammonium and nitrate by the above-ground seagrass tissue in this study were highest followed by epiphytes, below-ground seagrass tissue, with the lowest rates by plankton. Overall, the above-ground biomass dominated the resource allocation of ammonium, accounting for between 71 and 93% of the total resource, followed by epiphytes (9–17%) and the below-ground biomass (1–12%). Similarly, the above-ground seagrass tissue dominated the assimilation of nitrate accounting for 72–83% of the total resource in contrast to epiphytes (11–13%) and the below-ground tissue (5–15%). As with ammonium, assimilation of nitrate by plankton was negligible in this study. Complementing the findings of this study, Invers *et al*.^60^ reported that leaf tissue accounted for 60–87% of the total nitrogen assimilated by the temperate seagrass *Posidonia oceanica*. Similarly, the ammonium and nitrate uptake rates measured in this study are of the same magnitude as the rates reported for *Posidonia* and *Amphibolis* in the Adelaide coastal waters during summer^18^. In contrast, ammonium uptake rates reported by Alexandre *et al*.^27^ for leaves and roots of *Zostera noltii* were about 6 times the rates reported in this study. This could be attributed to the higher spike concentrations (~3.5 times higher than this study) used by Alexandre *et al*.^27^. On the other hand, nitrate uptake rates for leaves and roots reported by these authors were comparable to the rates observed here.

The fact that uptake of nitrogen by the above-ground seagrass tissue is more significant than by the below-ground seagrass tissue can be attributed to the habitat in which seagrasses thrive. Seagrasses inhabit shallow coastal areas often characterised by high ammonium concentrations in the sediment pore water in contrast to negligible concentrations of nitrate^13^. Actively growing seagrasses often take up most of the pore water nitrogen in the form of ammonium, whilst the leaves take up both ammonium and nitrate from the water column^23–25,61,62^. Leaves are therefore better adapted to take up nitrogen than roots, especially at low ambient concentrations^13,21^. This is further supported by the increased activity and concentrations of enzymes associated with the uptake of ammonium (glutamine synthetase) and nitrate (nitrate reductase) in the leaf tissues of *Zostera noltii* compared with root tissue, highlighting the significant role of leaves in nitrogen assimilation compared with roots^58^. In their comprehensive review, Hemminga *et al*.^43^ stated that although the majority of seagrasses grow in oligotrophic waters, the strategies adopted by seagrasses to conserve nutrients, such as resorption, are not well evolved. As a consequence, effective uptake of nutrients by leaves is an important strategy that seagrasses have adopted in order to maintain an adequate nutrient balance, especially in meadows where their distribution is patchy. The authors went on to conclude that the constraints imposed by the environment led seagrasses to favour this strategy over the development of an efficient nutrient conservation strategy. Complementing the findings of this study, Pedersen *et al*.^24^ recorded maximal leaf uptake rates to be 5–38 fold higher than the root-rhizome complex in *Amphibolis antarctica*. *Ruppia maritima*^63^, *Thalassia testudinum*^21^, *Thalassia hemprichii*^10^ and *Enhalus acoroides*^10^ are the only known seagrasses where root uptake dominates leaf uptake. Erftemeijer and Middleburg^10^ suggested that root uptake could potentially account for between 66 and 98% of the total nutrients taken up by tropical seagrasses *Thalassia hemprichii* and *Enhalus acoroides*.

The physiological interaction of the above-ground and below-ground seagrass compartment in nutrient acquisition depends on the concentration and the compartment exposed to the nutrient^63,64^. *Zostera* has been stated to adapt well to nitrogen-poor environments by taking up nitrogen from both the water column as well as sediments, by conserving nitrogen within the plant and by maintaining high growth rates despite low internal nitrogen reserves^61^. In their studies on *Zostera marina*, Thursby and Harlin^64^ reported that the uptake of ammonium by leaves was not influenced by the availability of ammonium to the roots. On the other hand, root uptake declined significantly when leaves were exposed to ammonium. Sub-tidal eelgrass in temperate regions of the world exhibits prolonged periods of low growth and high nutrient availability (e.g., winter, early spring and late autumn). It has been observed that during these periods, the plants incorporate surplus nutrients in the above-ground as well as below-ground biomass to offset the high nutrient demand associated with high growth under low ambient nutrient availability as seen during late spring and summer^65^. This is facilitated through luxury uptake mechanisms in seagrasses^66^ when ambient nitrogen concentrations are high. However, in seagrasses such as *Amphibolis*, surge or luxury uptake has been stated to be of little ecological relevance as they seldom encounter nitrogen-enriched ambient conditions^24^. However, that could not be said for *Zostera* that thrive in highly seasonal, low to high nutrient environments. Lee and Dunton^21^ also reported increased acquisition of nitrogen during summer and autumn in contrast to winter and spring. A study carried out in PPB (study site) and the adjacent Western Port revealed that interstitial water in the sediments was nitrogen limited during spring and summer^32,67^, with seagrasses resorting to leaf uptake. While studying uptake of nitrogen by *Zostera marina* from the external medium, Pedersen and Borum^50^ concluded that over 49% of the nitrogen requirements of the plants were met from the water column with the remaining 51% from the sediments. Short and McRoy^26^ proposed an ammonium uptake mechanism in *Zostera*, where the leaves monopolise the sporadic water column supply of nitrogen while maintaining a continuous assimilation of sedimentary nitrogen. These strategies in temperate seagrasses allow them to resort to leaf and/or root uptake under certain conditions are very significant in determining their ability to outcompete phytoplankton, epiphytes and other phototrophs by utilising small pools of bioavailable nitrogen in the water column and sediments more efficiently^68,69^.

In addition to direct impacts through shading, smothering or altering the sediment-water chemistry, epiphytes block active nutrient uptake sites on leaves^70^ thereby limiting availability of nutrients to seagrass. In some cases nitrogenous nutrients have been reportedly transferred from seagrass leaves to epiphytes and vice versa^71^. The epiphytes on seagrasses are known to take up ammonium and nitrate from the water column^72^. Fast growing epiphytes often outcompete seagrass under conditions of increased nitrogen availability^18,73–76^, eventually leading to a reduction in leaf production^77^. Specific uptake rates for ammonium and nitrate by epiphytes recorded in this study were significantly higher than the rates reported by Apostolaki *et al*.^78^, but comparable to the rates reported by Nayar *et al*.^18^. Although other studies report epiphytic nitrogen uptake rates to be several fold higher than for seagrass leaves^18,72,78,79^, this study recorded lower epiphytic uptake rates of about 0.6 times for ammonium and 0.87 times for nitrate than seagrass leaves. These results agree with the findings of Paling and McComb^52^ who reported significantly higher uptake rates for the seedlings of *Amphibolis antarctica* at higher nitrogen concentrations than Although the epiphytic nitrogen uptake rates reported in this study are low, epiphytes do play an important role as a sink for nitrogen, especially in the short term^79^. This study recorded 6–17% of the total ammonium and between 11–14% of the total nitrate resource assimilated by epiphytes. This is comparable to the assimilation of 28% by epiphytic algae on *Zostera marina*^26^.

The contribution of phytoplankton to the assimilation of ammonium or nitrate in this study was negligible, accounting for <0.05% of the total biological uptake. This is attributed to negligible phytoplankton biomass or standing crop. However, from a productivity perspective, Longmore *et al*.^80^ reported PPB to be a phytoplankton dominated system with the nutrient dynamics in the Bay closely related to phytoplankton growth. However, the CSIRO^81^ study reported algal biomass in the Bay to be strongly controlled by nitrogen concentrations at all times. Chlorophyll concentrations in the Bay were reported to be low at 1–20 mg.m^−3^, and at the bottom of the range when compared to temperate eutrophic European and North American estuaries. This was attributed to nitrogen limitation leading to low nitrogen to phosphorus ratios. Nitrogen to phosphorus ratios in PPB are low due to (1) phosphorus inputs being relatively higher than nitrogen inputs, and higher than what can be assimilated biologically, and (2) depletion of inorganic nitrogen by denitrification leading to most of the inorganic nitrogen being lost to the atmosphere as nitrogen gas. CSIRO^81^ went on to suggest that with most of the inorganic nitrogen depleted, the organic fraction left behind accounted for well over 90% of the total nitrogen in the Bay. This limitation of inorganic nitrogen was therefore concluded to limit phytoplankton biomass in PPB^82^.

An important consideration for *in situ* studies using benthic chambers to quantify nutrient uptake rates of seagrasses, epiphytes and phytoplankton at environmentally realistic levels of nitrogen, is the risk of running into nutrient depletion over the course of the incubation. This is a major concern in scenarios replicating low nutrient environments where the concentrations of nutrients spiked into the chambers are low (i.e. close to ambient levels). Depletion of nutrients prematurely could lead to significant underestimation of uptake rates, with potential nutrient down- or up-regulation mechanisms remaining undetected^49^. However, this study was conducted under nutrient sufficient conditions, as demonstrated from the amount of nutrient resource left at the end of a 2 hour incubation. This was determined to be 24.6 ± 5.3% for ammonium and 14.2 ± 4.9% (mean ± standard error; n = 6) of the total resource for nitrate uptake experiments, a confirmation that the system was not totally depleted of nutrients during the course of incubation.

As with nutrient uptake mechanisms, the processes of assimilation and translocation of nutrients are adaptation strategies employed by seagrasses to maximise the nutrients available for growth. It is reasonable to expect higher rates of biological production to be accompanied by increased demand and assimilation of nutrients in marine environments. In spring and early summer in temperate oligotrophic seagrass meadows, the rate of growth supersedes the availability of nutrients for uptake^83^. Under conditions of nutrient limitation and high growth, two strategies widely adopted by terrestrial plants to conserve and rationalise nutrient use are limiting losses through leaching and translocation of nutrients from senescing tissues to new growth, thereby contributing to high nutrient use efficiency^84–86^. Borum *et al*.^65^ and Pedersen and Borum^61^ concluded that losses of nutrients due to leaching were relatively insignificant, in contrast to the translocation process^42,61,65,87^. According to these authors, leaching never exceeded 10% of the total nutrient losses in *Zostera marina*.

Alcoverro *et al*.^44^ hypothesised three strategies adopted by *Posidonia oceanica* during the growth cycle to meet its nutritional demand. These strategies involved: acquisition of nutrients from external sources, such as the water column or the pore water; recycling internal nutrient pools, including translocation from one organ to the other; and lastly utilisation of stored nutrients from the leaf tissue. Under high ambient nitrogen concentrations, seagrasses are known to increase their nitrogen uptake rates, often to a point where uptake rates surpass nitrogen requirements of the plant. In such instances the excess nitrogen is stored internally as amino acids or proteins to meet high nitrogen demands coinciding with periods of high production^60,88,89^. Next to uptake or assimilation of nutrients, translocation has been stated to be an important process in meeting the nutrient requirements of seagrasses^13^.

Based on the evidence in the existing literature, we hypothesise two major internal nutrient transfer processes in seagrasses. One is an adaptive strategy and the other is a conservation strategy. The nutrient translocation process is purely an adaptation strategy, where the compartment (above-ground or below-ground) exposed to higher concentrations of the nutrient takes up the nutrient, and shunts it to the opposite compartment for storage or to meet the metabolic or growth needs. As seen in this study the translocation of nutrients from the below-ground biomass (rhizomes and roots) to the above-ground biomass (leaves and shoots) is more significant than the other way around. Only a small percentage of the nutrients taken up by the above-ground biomass is translocated to the below-ground biomass, with most instead retained in the above-ground biomass to meet growth needs. The nutrient re-translocation process on the other hand is a conservation strategy whereby nutrients are mobilised from a senescing organ or tissue to an actively growing tissue where they undergo a transition from being a sink to a source of nutrients^65,90^. This has been regarded to be the main mechanism by which conservation of nutrients by seagrass under low ambient nutrient conditions is accomplished^44^. Borum *et al*.^65^ found that up to 90% of the nitrogen from the old leaves in *Zostera marina* was recovered in other plant parts through the process of re-translocation.

This study has demonstrated the uptake of nutrients by the above-ground and below-ground seagrass biomass from the water column and pore water, and the subsequent translocation to the opposite compartment. The assimilation by the above-ground biomass of both ammonium and nitrate from the water column (97–100% ammonium and 94–97% nitrate) dominated assimilation by the below-ground biomass from the pore water (68–82% ammonium and 8–89% nitrate). The quantum of assimilated nutrients translocated from the below-ground to above-ground biomass (18–32% for ammonium and 11–19% for nitrate) thus surpassed the translocation from the above-ground to below-ground biomass (0–3% for ammonium and 3–6% for nitrate). This conforms to the findings of other researchers who report translocation to, and retention within, the more actively growing part of the plant. In a study on *Zostera marina*, Borum *et al*.^65^ found that translocation and internal recycling of nitrogen to new leaves accounted for up to 69% of the total nitrogen gained. Similarly, Alcoverro *et al*.^44^ reported nitrogen uptake of 60% and re-translocation of 40% in *Posidonia oceanica*. The values reported by these authors and the results of the current study were higher than the 15% reported for seagrasses by Stapel and Hemminga^42^. These results, however, differ from the findings of Alexandre *et al*.^27^ who reported less than 1% of the inorganic nitrogen incorporated by the leaves or roots being translocated to the other compartment in *Zostera noltii*. Similar observations were made by Vonk *et al*.^48^ in *Thalassia hemprichii*, *Halodule uninervis* and *Cymodocea rotundata*, where less than 1% of the nitrogen taken up by the leaves was translocated to the roots in short incubations lasting 1 hour. On the contrary, 8–20% of the nitrate incorporated by the roots were translocated to the leaves, in close agreement with the findings of this study.

Thursby and Harlin^64^ reported that nutrient concentration and the compartment (above-ground or below-ground) that is exposed to the nutrients influenced the uptake and translocation processes. In their studies on *Zostera marina*, leaf to root translocation (basipetal) of ammonium dominated root to leaf translocation (acropetal). However, no interactions between leaves and roots in nitrogen uptake were observed for shorter incubations lasting 1–5 hours for *Zostera noltii*^27^, *Zostera marina*^26^, *Thalassia hemprichii*^23^ and *Phyllospadix torreyi*^25^. Izumi and Hattori^91^ concluded that at the end of a 24 h incubation of the leaves of *Zostera marina* with ^15^N labeled ammonium, the enrichment of the root-rhizome tissue was similar to that in the leaf tissue, suggesting a rapid translocation of ammonium from the leaves to the below-ground tissues (basipetal). However, when the roots were exposed to the tracer in the same study, the enrichment of ^15^N in the leaves was a magnitude lower than that of the below-ground biomass. These results differ from the findings of this study where acropetal translocation dominated basipetal translocation of both ammonium and nitrate in short-term incubations of 2 hours. In their review, Hemminga *et al*.^87^ suggested that, as a significant quantum of the biomass production occurs in the above-ground compartment, it would be reasonable to expect acropetal translocation when nitrogen uptake was dominated by the below-ground compartment; that is in close agreement with the findings of this study when the ‘pore water’ compartment was spiked. Therefore, the exposure of the below-ground biomass to high nitrogen concentrations resulted in significant enrichment, largely attributed to direct uptake of labeled nitrogen by the roots of *Zostera nigricaulis*. The translocation of inorganic nitrogen from the below- to the above-ground biomass was stated to be an energy positive process, where the required energy is supplied by photosynthesis^91^. The authors hypothesised that light-dependent nitrogen translocation may be facilitated by one of the following processes: (1) active translocation through the vascular system; (2) active transport across the cell membranes; and (3) the supply of carbon skeletons to the roots, and their transport across the cell membranes and translocation through the vascular system. In a study that compared nutrient dynamics in *Posidonia coriacea* and *Zostera tasmanica*, two dominant seagrass species in Success Bank, Western Australia, Walker *et al*.^92^ stated that the vegetative growth of *P. coriacea* was more nitrogen limited than *Z. tasmanica*, especially during periods coinciding with high growth and low ambient nutrients. The authors went on to suggest that acropetal translocation of nutrients ensured that the growing tissues were not nutrient limited and that growth could be sustained, a phenomenon more apparent in *Z. tasmanica* than in *P. coriacea*.

Lee and Dunton^21^ concluded that seagrasses allocate more biomass into the below-ground tissues under conditions of low sediment nitrogen availability, as an adaptation to increase surface area of roots, and thereby enhance nitrogen uptake. Conversely, an increase in ambient sediment nitrogen concentration is mirrored by an increease in the above-ground biomass to enhance carbon fixation to meet increased biological production. Similar conclusions were drawn by Alexandre *et al*.^27^ on increased root uptake in *Zostera noltii*, although other researchers have concluded that direct uptake of nitrogen by the roots is negligible in other species^13,23,43^. These studies complement the findings of this study, where the evidence shows that acropetal translocation may be prevalent in certain seagrass species as an adaptive strategy under certain growth and environmental conditions.

### Conclusions and management implications

*Zostera nigricaulis* in PPB demonstrated a clear preference for ammonium over nitrate as the source of nitrogen. This result is comparable to other species of seagrasses, as the plant requires far less energy to transform the reduced source of inorganic nitrogen (ammonium) into organic nitrogen than the oxidised forms (nitrate or nitrite). However, the presence of epiphytes and phytoplankton in the seagrass community adds another level of complexity to the biotic uptake processes, as epiphytes and phytoplankton (algae) are opportunistic in the uptake of nitrogen and often out-compete seagrasses. Seagrasses, like algae, can adopt strategies, such as luxury uptake, and uptake using above-ground biomass in nitrogen limited systems. In PPB, the above-ground biomass of *Z. nigricaulis* was more efficient in uptake and assimilation of inorganic nitrogen than the below-ground biomass, with the seagrass translocating nitrogen resources from one compartment to the other. The study demonstrated: (a) that both the above- and below-ground biomass assimilated nitrogen, depending on its availability either in the water column or in pore water; and (b) that the above-ground biomass, especially the leaves, played a critical role in the nitrogen assimilation process. Whilst the below-ground biomass assimilated nitrogen from the pore water, the plant did not utilise the below-ground biomass as a reserve to store this nitrogen pool. Unlike most other seagrasses, this study demonstrated a novel outcome, namely that acropetal translocation dominated over basipetal translocation. As the study was conducted *in situ* under environmentally realistic conditions, the results have significant application in the development of whole ecosystem models to enable ecosystem managers to better understand ecosystem process and help develop policies and strategies to better manage seagrass ecosystems in PPB. However, further studies are required to further our understanding on uptake at concentrations a magnitude lower than the levels used in this study and comparing results of short term incubation with longer term incubations.

## Data Availability

Data used in this manuscript will be available to the public.
